# Wideband Dual-Polarized PRGW Antenna Array with High Isolation for Millimeter-Wave IoT Applications

**DOI:** 10.3390/s25113387

**Published:** 2025-05-28

**Authors:** Zahra Mousavirazi, Mohamed Mamdouh M. Ali, Abdel R. Sebak, Tayeb A. Denidni

**Affiliations:** 1 Department of Electrical and Computer Engineering, Concordia University, Montreal, QC H3G 1M8, Canada; abdo@ece.concordia.ca; 2 Electrical Engineering Department, Assiut University, Assiut 71515, Egypt; mohamed.ali@ieee.org; 3 Institut National de la Recherche Scientifique-Energie (INRS), Montreal, QC H4V 1B7, Canada; tayeb.denidni@inrs.ca

**Keywords:** dual polarization, magneto-electric (ME) dipole, internet of things (IoT), printed ridge gap waveguide (PRGW), millimeter-wave (mm-wave)

## Abstract

This work presents a novel dual-polarized antenna array tailored for Internet of Things (IoT) applications, specifically designed to operate in the millimeter-wave (mm-wave) spectrum within the frequency range of 30–60 GHz. Leveraging printed ridge gap waveguide (PRGW) technology, the antenna ensures robust performance by eliminating parasitic radiation from the feed network, thus significantly enhancing the reliability and efficiency required by IoT communication systems, particularly for smart cities, autonomous vehicles, and high-speed sensor networks. The proposed antenna achieves superior radiation characteristics through a cross-shaped magneto-electric (ME) dipole backed by an artificial magnetic conductor (AMC) cavity and electromagnetic bandgap (EBG) structures. These features suppress surface waves, reduce edge diffraction, and minimize back-lobe emissions, enabling stable, high-quality IoT connectivity. The antenna demonstrates a wide impedance bandwidth of 24% centered at 30 GHz and exceptional isolation exceeding 40 dB, ensuring interference-free dual-polarized operation crucial for densely populated IoT environments. Fabrication and testing validate the design, consistently achieving a gain of approximately 13.88 dBi across the operational bandwidth. The antenna’s performance effectively addresses the critical requirements of emerging IoT systems, including ultra-high data throughput, reduced latency, and robust wireless connectivity, essential for real-time applications such as healthcare monitoring, vehicular communication, and smart infrastructure.

## 1. Introduction

The Internet of Things (IoT) has revolutionized the fifth generation (5G) of communication by enabling the interconnection of numerous devices, facilitating applications such as smart homes, smart cities, efficient transportation systems, and augmented/virtual reality (AR/VR) [1,2,3]. As the number of connected devices increases, there is a growing demand for high data rates and low latency to support these applications [4,5,6,7]. Integrating millimeter-wave (mm-wave) technology into the 5G spectrum plays a critical role in meeting these demands. Operating in the 30 GHz to 300 GHz frequency range, mm-wave technology offers a broader bandwidth than traditional communication systems, allowing for higher data throughput and reduced congestion [8,9].

These advantages are crucial for the next-generation IoT networks, where antenna arrays, such as the one proposed in this work, can significantly enhance performance by enabling efficient communication with high data rates and minimal interference [10,11,12,13]. By utilizing this advanced technology, wireless networks are better equipped to meet the evolving requirements of IoT applications.

One of the primary advantages of mm-wave frequencies is the significant increase in data transmission speeds. The wider bandwidth available at mm-wave frequencies allows for much higher data rates and increased network capacity, making them ideal for the high-demand environment of IoT applications. Additionally, mm-wave technology offers a substantial reduction in network latency, which is critical for real-time interactions in IoT applications such as healthcare, automotive, and smart cities [14,15,16]. The expanded bandwidth dramatically enhances the capacity of IoT networks, allowing for seamless data sharing among a large number of devices, which is essential as IoT grows. Ensuring connectivity for many devices simultaneously, while maintaining high-speed communication and quality of service, is key to unlocking the full potential of the IoT [17,18,19]. To support this, effective antenna designs are crucial, particularly for addressing the unique propagation challenges of mm-wave frequencies, including increased signal loss and sensitivity to obstacles. Researchers have proposed promising mm-wave antenna designs tailored for high-speed 5G connections and IoT applications [11,20,21,22].

**Figure 1:**
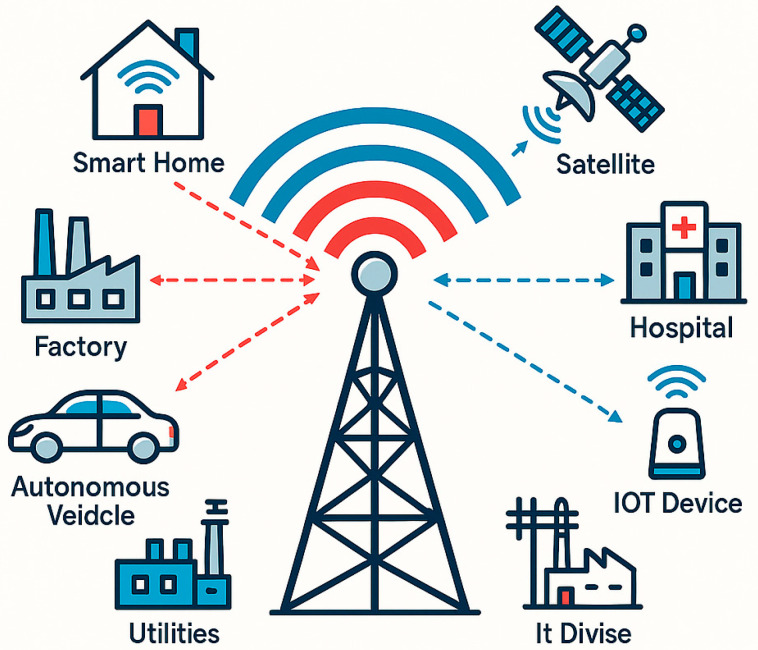
Diagram of a dual-polarized antenna in an IoT network: The antenna at the core sends and receives signals vertically (red) and horizontally (blue). This figure was created with the assistance of an AI tool (ChatGPT 4.5, OpenAI, San Francisco, CA, USA).

To tackle these propagation challenges, modern antenna designs focus on compactness, higher gains, and broader bandwidths, often integrating Multiple Input Multiple Output (MIMO) technologies [23,24]. Dual-polarized antennas are particularly effective in MIMO systems, improving multipath interference and channel capacity by offering two orthogonal polarizations. As shown in Figure 1, this advanced antenna technology meets the demands of mm-wave IoT applications by utilizing the same frequency band for both transmission and reception, minimizing signal interference and enhancing data transmission capacity, critical for maintaining reliable communication in congested IoT networks. Figure 1 demonstrates how vertical and horizontal polarizations work together to enhance communication. Applications such as smart homes, autonomous vehicles, and other IoT systems rely on the continuous exchange of information facilitated by these sophisticated antennas.

Integrating dual-polarized antennas into IoT infrastructure greatly enhances the communication capabilities of smart devices, allowing for rapid and reliable data exchange and moving us closer to a fully connected world [25,26]. These antennas play a crucial role in supporting large numbers of devices without signal interference, making them vital for expanding 5G networks and developing smart technologies. High isolation in dual-polarized antennas is a key performance metric for both IoT and mobile communication applications, as it significantly reduces mutual coupling and cross-polarization interference between ports. In dense IoT environments, where a large number of devices operate simultaneously and require robust wireless links, insufficient isolation can result in signal degradation, reduced channel capacity, and unreliable data transmission [27,28,29,30,31].

Recently, researchers have explored various dual-polarized antenna designs for mm-wave technologies, which are essential for advancing IoT applications. However, many existing designs face challenges related to limited bandwidth or large dimensions, which can impede the performance and scalability of IoT networks. For instance, popular patch antennas with single-mode operation often exhibit a relatively narrow bandwidth, typically around 6% [32,33,34], limiting their ability to support high-speed communication in dense IoT environments. To address this limitation, several feeding methods, such as U-shaped slots [35], L-probes [36,37], and substrate-integrated waveguides (SIW) [38,39,40], have been explored, achieving bandwidths of up to 15%, 23%, and 28%, respectively. However, while these designs offer broader bandwidths, they often result in lower gains, typically around 10 dBi, which affects their ability to maintain reliable connections in large-scale IoT networks. Low-temperature co-fired ceramic (LTCC) technology has been used to achieve higher gains, but this approach leads to larger antenna sizes, often requiring more than ten layers [41,42], making it unsuitable for antenna array designs in compact IoT devices, where size and integration are critical factors.

The magneto-electric (ME) dipole antenna, which combines electric and magnetic dipoles based on complementary source theory, is gaining popularity in IoT systems for its wide impedance bandwidth, stable gain, and symmetrical radiation pattern [43,44]. The ME-dipole design typically incorporates single-layered resonators, shorted vias for the magnetic elements, and patches for the electric components, all positioned on the same substrate. This structure is particularly effective for high-frequency applications, offering broad bandwidth and reliable radiation characteristics that are crucial for ensuring seamless communication across IoT devices. Various feeding techniques, including L-shaped and T-shaped configurations, have optimized performance, making the ME dipole antenna a promising choice for advanced IoT wireless communication systems [41,45,46,47]. Wideband dual-polarized ME dipole antennas, fabricated using LTCC technology and different feeding mechanisms, have been discussed in [41,45]. However, the isolation levels in these designs are generally below 18 dB as described in [41,45]. In contrast, studies in [46] (fed by SIW) and [47] (fed by microstrip line) have presented high-isolation dual-polarized aperture-coupled ME-dipole antennas, achieving isolation levels of 40 dB and 35 dB, respectively. While these designs offer impressive port isolation, their bandwidths remain relatively narrow, around 21% and 16.7%, which limits their applicability in high-density IoT networks where broad bandwidth and high isolation are crucial for maintaining reliable performance across numerous devices.

This growing interest in advanced guiding structures and antenna technologies is driven by the increasing demands of IoT systems for efficient, high-performance communication, where dual-polarized antennas and novel waveguide designs play a crucial role in addressing these challenges. Technologies like substrate-integrated waveguides (SIW) or microstrip lines can face significant transmission line losses when used in large-scale antenna arrays, especially at millimeter-wave frequencies. This can lead to reduced radiation efficiency, which is a critical issue for applications that require high-speed communication, such as IoT systems. As the demand for high-frequency communication increases, there is a need for novel guiding structures that can minimize radiation and material losses at higher frequency bands. Ridge gap waveguide (RGW) and printed RGW (PRGW) are emerging technologies that show great potential in addressing these challenges, as highlighted in various studies [48,49,50,51]. Recently, some works have focused on designing dual-polarized antennas based on RGW and PRGW technologies [52,53,54]. For example, the design introduced in [42] features a dual-polarized antenna array with a 3D-printed RGW feeding network, demonstrating satisfactory performance. However, the antenna structure remains bulky, and the manufacturing process presents challenges. In contrast, the dual-polarized ME dipole antenna array described in [53], utilizing PRGW technology, employs a dual-polarized fork-shaped microstrip line and integrates a dual-polarized split-ring resonator (SRR) to enhance gain. This SRR functions as a meta-lens to reshape radiated spherical waves into plane waves for both polarizations.

While the design in [53] achieves a notable gain of 16 dBi, its impedance bandwidth is limited to 22%, and the addition of a split-ring resonator (SRR) lens results in increased size and fabrication complexity, potentially limiting its suitability for compact systems. Another recent study [54] presents a compact dual-polarized ME-dipole antenna fed by PRGW at 30 GHz, with a bandwidth of 23.4% and a gain of 10.5 dBi; however, the port isolation is about 20 dB, which may not be sufficient for interference-free operation in dense environments. SIW-fed ME-dipole arrays reported in [46] target higher mm-wave bands around 60 GHz, achieving bandwidths of 21–22% and isolation above 15 dB, though often with increased structural complexity and less compatibility with low-profile PCB integration.

Building on the outlined challenges, such as limited bandwidth, low gain, and inadequate isolation, this work introduces a novel solution to address these issues for millimeter-wave IoT applications. These limitations can affect the performance of advanced communication networks, where efficiency and reliability are essential. To address these challenges, this work presents a dual-polarized ME dipole antenna array designed to achieve high gain and wide impedance bandwidth in the millimeter-wave spectrum. By utilizing innovative PRGW technology, the design significantly enhances isolation and radiation efficiency, which are critical for ensuring reliable communication in dense network environments typical of IoT applications. The antenna features a 2 × 2 cross-shaped ME dipole array, optimized with an artificial magnetic conductor (AMC) to improve gain. The design achieves an impressive impedance bandwidth of 24% across the frequency range of 26.7–33.7 GHz, with peak performance at 30 GHz. Additionally, it provides high isolation levels exceeding 40 dB between dual polarizations, ensuring minimal interference. The antenna also delivers a consistent gain of approximately 13 dBi ± 0.5 dBi across the operating band, making it a robust solution for the high-speed, low-latency demands of IoT networks.

The structure of the paper is organized as follows: Section 2 details the geometric configuration of the designed dual-polarized antenna array and the innovative PRGW feed network. Section 3 discusses the design and operational principles, including unique subarray configurations and dual-mode feeding techniques. Section 4 provides fabrication results and performance evaluations, focusing on the robust functionality of the proposed antenna array. The conclusion is included in Section 5, mentioning a summary of the findings, while focusing on the potential of the antenna to improve the efficiency and throughput of modern wireless communication systems.

## 2. Dual-Polarized ME-Dipole Antenna Array Configuration

**Figure 2:**
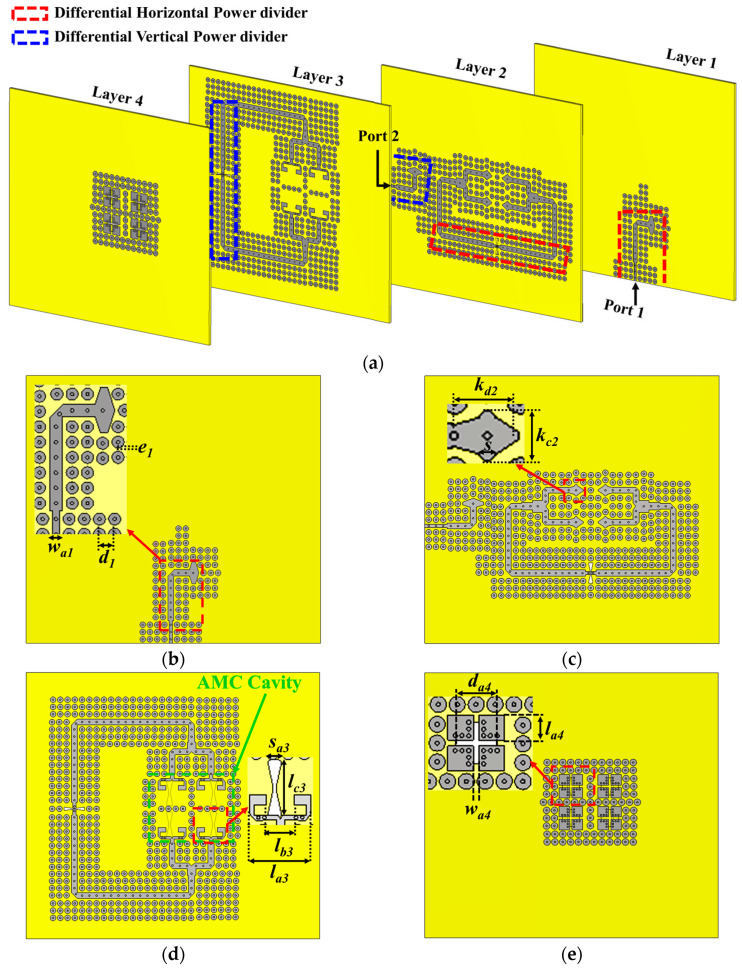
Geometry of the designed dual-polarized ME-dipole antenna array. (**a**) A 3-D view and top views of (**b**) layer 1, (**c**) layer 2, (**d**) layer 3, and (**e**) ME-dipole antenna.

The geometric configuration of the proposed dual-polarized ME-dipole antenna array and its feed networks, optimized for IoT millimeter-wave applications, is illustrated in Figure 2a, with detailed geometric parameters provided in Table 1.

The design incorporates four layers of Rogers RT6002 substrates (ε_r_ = 2.94) with a thickness of h_s_ = 0.762 mm.

A 2 × 2 ME-dipole antenna array, enclosed by electromagnetic bandgap (EBG) unit cells, is positioned on the topmost substrate layer. This configuration supports dual-polarized operation, essential for high-density IoT environments requiring robust and reliable connectivity. Each ME-dipole antenna includes vertical and horizontal electric planar dipoles (E-dipoles), implemented through four uniform square-shaped metallic patches printed on the upper surface, as shown in Figure 2b.

These patches connect to the substrate ground plane via five metallic vias near the inner corners of each patch. Vertical and horizontal magnetic dipoles (M-dipoles) are realized using a cross-shaped aperture that spans the ground plane and the patch surface. This aperture, comprising vertical and horizontal rectangular segments, serves as a magnetic dipole and is surrounded by plated vias to enhance isolation and reduce interference, critical features for IoT systems operating in crowded spectral environments.

To improve the antenna gain, an artificial magnetic conductor (AMC) surface, composed of mushroom-shaped EBG structures, surrounds the ME-dipole array, as illustrated in Figure 2e. The AMC surface is designed to mitigate surface wave losses and enhance radiation efficiency, meeting the stringent requirements of IoT devices operating at millimeter-wave frequencies. The 2 × 2 dual-polarized ME-dipole antenna array is excited via an AMC-walled cavity located in the third layer, comprising four distinct AMC cavities. This cavity is driven by two orthogonally placed low-loss PRGW lines situated in the first and second substrate layers. Port 1 supports vertical polarization (H-Pol), while Port 2 is dedicated to horizontal polarization (V-Pol). The PRGW lines are constructed using periodic circular patches forming mushroom-like EBG unit cells, which are key to maintaining low transmission losses.

Previous studies [55,56] have demonstrated the effectiveness of EBG structures in tuning the operating bandwidth of PRGW lines by leveraging their bandgap properties. In this design, the EBG unit cells feature an air gap (h_gap_ = 0.254 mm), creating a band-stop frequency range of approximately 23 to 40 GHz [54]. This configuration not only ensures high performance but also aligns with the stringent requirements of IoT systems, such as wide bandwidth, high isolation, and low interference, enabling efficient and reliable wireless communication in the millimeter-wave spectrum.

## 3. Design Guideline and Operating Principle

### 3.1. Subarray ME-Dipole Antenna

The 2 × 2 subarray ME-dipole antenna, designed for stable radiation performance across a wide frequency range, features a distinctive configuration. The grounded patch pairs on the upper surface of the top substrate act as electric dipoles (E-dipoles), while the apertures between these patches function as magnetic dipoles (M-dipoles), as illustrated in Figure 2e. The E-dipoles feature a radiation pattern that forms an 8-shaped pattern on the E-plane and an O-shaped pattern on the H-plane, and the M-dipoles exhibit complementary patterns. By arranging these dipoles orthogonally and exciting them simultaneously with carefully calibrated amplitude and phase, the antenna achieves a symmetrical, unidirectional radiation pattern with low back radiation. This characteristic is particularly advantageous for minimizing interference and enhancing signal clarity in dense IoT environments. The integration of these dipoles into a subarray configuration further elevates the antenna performance.

The design specifics of the individual ME-dipole are detailed in [54]. Excitation of the ME-dipole antenna is facilitated by an artificial magnetic conductor (AMC) cavity, highlighted within the green box in Figure 2d. This cavity is meticulously designed to support equivalent excitation modes for both vertical and horizontal polarizations, a key requirement for modern dual-polarized systems. The square AMC cavity, with dimensions of approximately 32 × 32 mm^2^, is tailored to excite the TM120 mode for vertical polarization and the TM210 mode for horizontal polarization. This dual-mode excitation optimizes the antenna operational efficiency across both polarization states.

**Figure 3:**
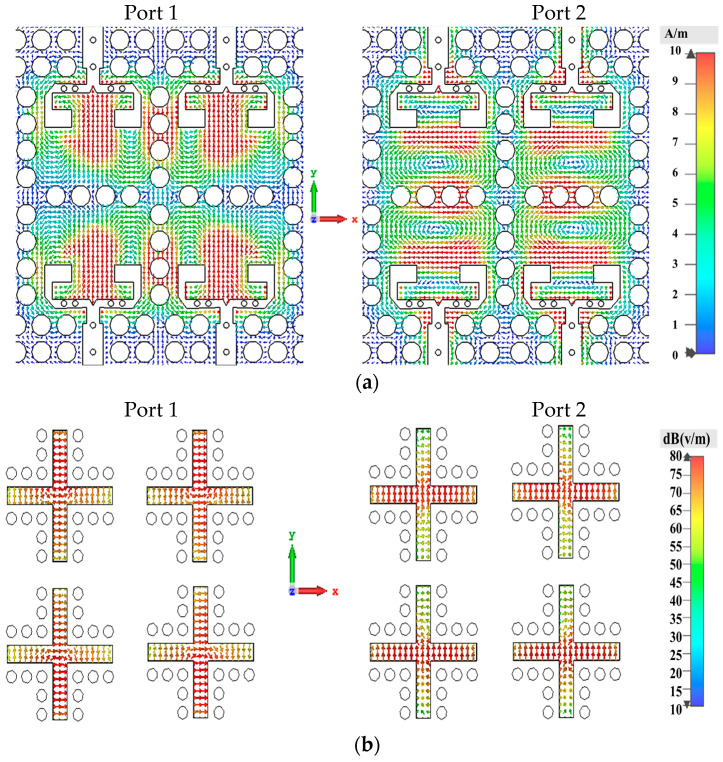
The dual-polarization excitation process of the ME-dipole antenna. (**a**) The magnetic field distributions in the cavity and (**b**) electric field distributions in the excited slots for Ports 1 and 2.

Figure 3 illustrates the distribution of magnetic fields within the square cavity and the corresponding electric fields in the slots for the TM120 and TM210 modes, activated by inputs at Port 1 and Port 2, respectively. This dual-mode capability underscores the ability of the antenna to support distinct modes of operation, highlighting its versatility and suitability for IoT applications in millimeter-wave communications.

### 3.2. Differential Feeding Network Design

**Figure 4:**
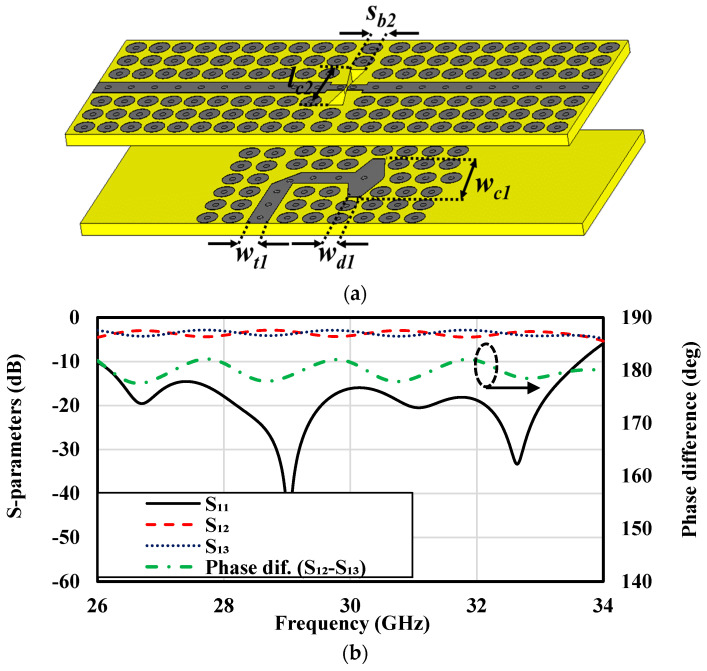
The bowtie-shaped coupled power divider: (**a**) configuration and (**b**) simulated S-parameters and phase differences.

To excite the cavity effectively, a differential feeding network is employed, consisting of two power dividers placed across separate substrate layers and coupled via bowtie-shaped slots, as shown in Figure 2b,c. These power dividers are driven by two orthogonal feeding structures designed to distribute power efficiently to the antenna elements. Each structure is implemented using PRGW lines, connected to the input ports located on the first and second layers. This arrangement ensures sufficient isolation between horizontal and vertical polarizations, which is critical for achieving efficient antenna operation and producing the desired radiation pattern with minimal polarization interference. Power is transferred from the input ports to the power dividers through bowtie-shaped coupling slots, each approximately λ/2 in length, as illustrated in Figure 4a. The primary function of the power divider is to evenly split the power while maintaining a consistent 180° phase difference between the outputs. This design approach results in a stable, broadside radiation pattern with low cross-polarization levels. Additionally, the element spacing is minimized to less than the free-space wavelength (λ_0_), enhancing the antenna’s overall performance. To ensure proper coupling and impedance matching, the input line of the power divider is terminated with a matching stub, as indicated in Figure 4a. The bowtie-shaped coupling slot integrates two opposing PRGW lines, guaranteeing equal power division and the necessary 180° phase difference at the output ports. Simulated S-parameters, presented in Figure 4b, validate the effectiveness of this design. The results demonstrate an excellent matching level of better than −15 dB across a 23% relative bandwidth spanning 26.4 to 33.3 GHz. Furthermore, the power divider ensures equal power distribution to the output ports (−3 dB) while maintaining the critical 180° phase difference, underscoring the reliability of the proposed feeding network for dual-polarized millimeter-wave applications.

### 3.3. In-Phase Feeding Network Design

**Figure 5:**
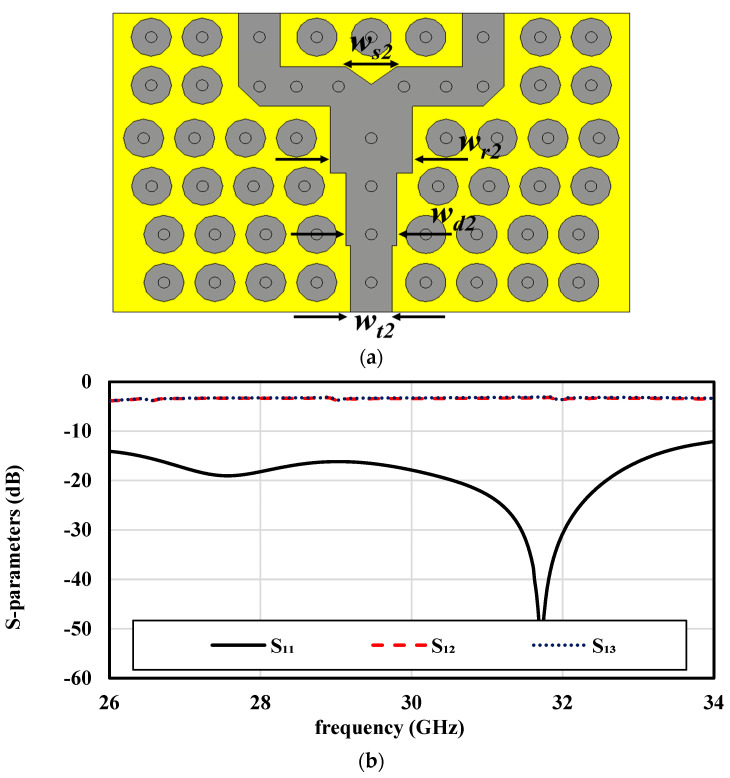
The in-phase power divider: (**a**) configuration and (**b**) simulated scattering parameters.

**Figure 6:**
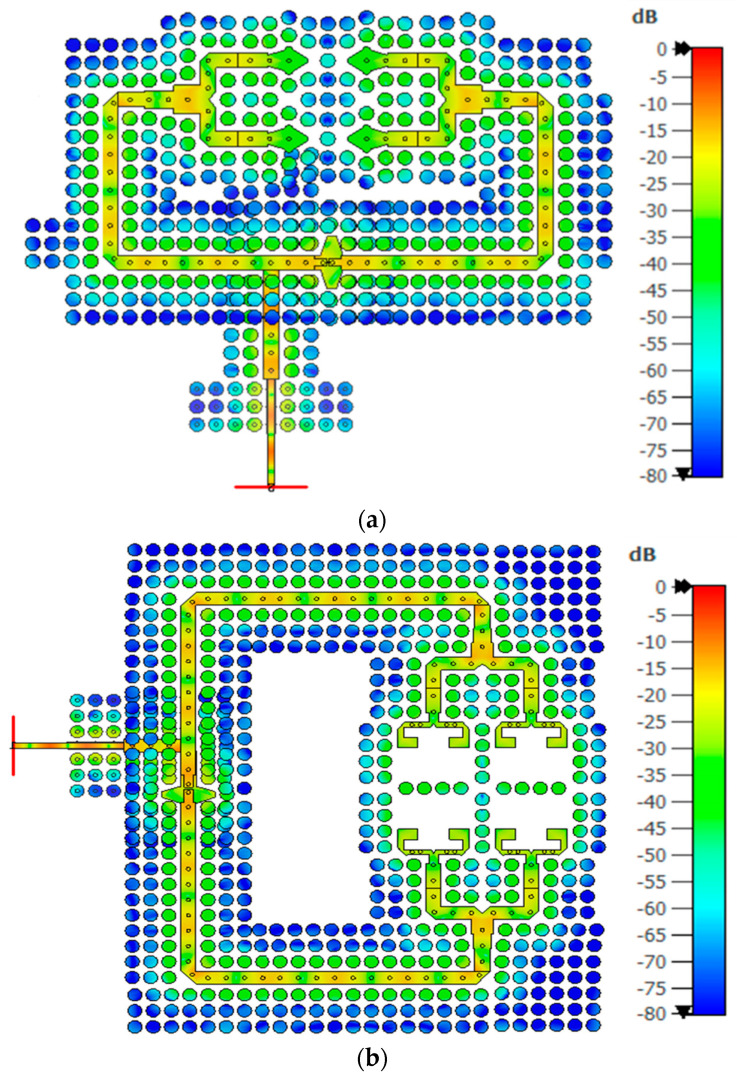
Surface current distributions at 28 GHz in the proposed power divider: (**a**) from input port 1 to the power divider located in the second layer and (**b**) from input port 2 to the power divider located in the third layer.

To ensure efficient power radiation to the 2 × 2 ME-dipole antenna through the AMC cavity, two 1 × 2 in-phase power dividers are implemented in the second and third layers, as illustrated in Figure 2c,d. Figure 5a details the design, featuring two transformers of identical lengths (l_tp_ = 5 mm) but varying widths (W_t1_ and W_t2_) and a small triangular slot for achieving deep impedance matching. The simulated S-parameters, shown in Figure 5b, demonstrate uniform power distribution (−3 dB) with a consistent 0° phase difference and excellent matching levels exceeding −20 dB across the entire operating bandwidth. This optimized power division is crucial for achieving the reliability and low interference needed in IoT millimeter-wave applications. Figure 6 provides the surface current distributions at 28 GHz within the power dividers, highlighting their effectiveness. Figure 6a shows how energy from input Port 1 is efficiently coupled into the second-layer divider and evenly distributed to its outputs, while Figure 6b illustrates a similar performance for Port 2 and the third-layer divider. The PRGW structure effectively confines energy, preventing undesired losses and ensuring reliable, efficient energy transmission, critical for IoT-focused communication systems where performance and precision are key.

### 3.4. Operating Mechanism

**Figure 7:**
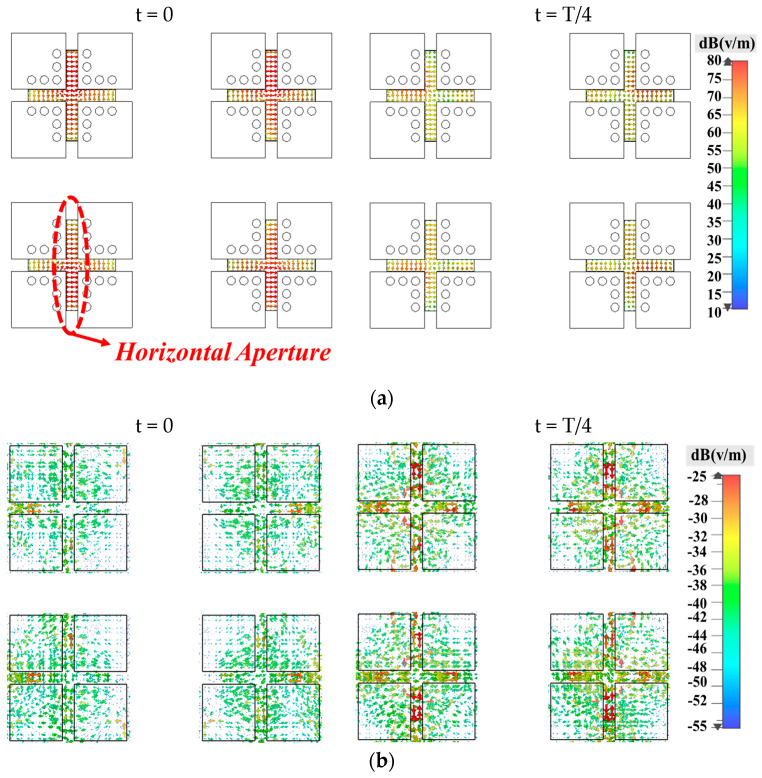
Operating mechanism of the proposed dual-polarized antenna with port 1 excited. (**a**) Electric field distribution across the radiating aperture at t = 0 and T/4 and (**b**) current distribution on the antenna surface at t = 0 and T/4.

Figure 7 illustrates the working principle of the dual-polarized antenna when Port 1 is excited at a center operating frequency of 28 GHz. This figure showcases two key aspects of antenna behavior, including the electric field distribution across the radiating aperture and the current distribution across the antenna surface, both observed at t = 0 and t = T/4, where T represents the period of the operating frequency. At t = 0, the antenna demonstrates a strong electric field at the horizontal radiating aperture and a low current distribution on the patches, indicating significant excitation of the magnetic dipole. This phase is characterized by the maximum electric field density and minimal current density, aligning with the magnetic components peak activation. At t = T/4, there is a noticeable shift with the electric field weakening and the current density peaking at the patches’ outer edges, highlighting the electric dipole’s primary excitation. Similarly, when Port 2 is excited, a similar operation can be expected, but with a key difference in the orientation of the electric and magnetic dipoles. In this case, both dipoles are oriented perpendicularly compared to their orientation when Port 1 is excited. Thus, it is not shown here. This behavior highlights that the proposed antenna can alternately excite the electric and magnetic dipoles with a 90° phase difference and in perfectly orthogonal orientations. This special arrangement ensures that the antenna operates efficiently as a complementary system, allowing it to maintain a consistent and directional radiation pattern regardless of which port is excited.

### 3.5. Effect of AMC Surface on Gain and Radiation Pattern

**Figure 8:**
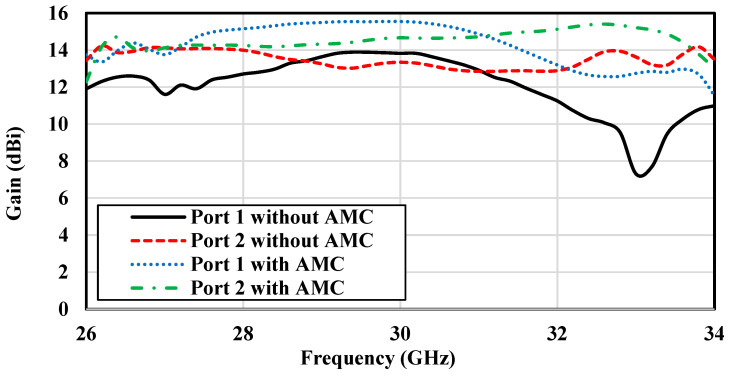
Simulated gain of the proposed dual-polarized antenna without and with the presence of the AMC.

**Figure 9:**
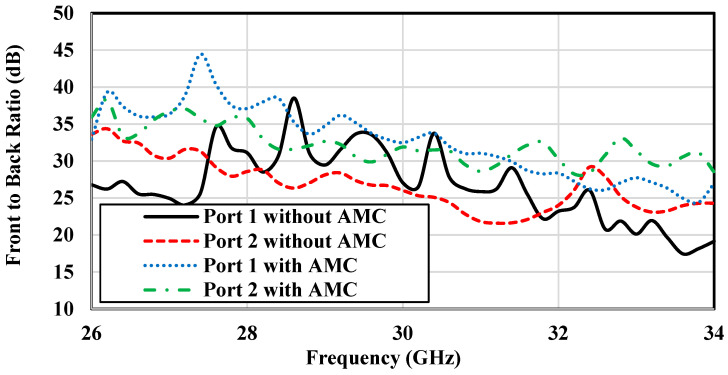
Simulated front-to-back ratio of the designed dual-polarized antenna without and with the presence of AMC.

As previously mentioned, an array of mushroom-shaped EBG unit cells is deployed around the ME-dipole antenna to serve as an AMC surface to enhance the gain of the proposed antenna. The impact of this AMC surface on the antenna performance is analyzed in this section. Figure 8 and Figure 9 show that the presence of the AMC surface plays a significant role in enhancing the gain and improving the front-to-back ratio of the antenna for both ports. The incorporation of the AMC surface leads to a gain increase of approximately 2 dB and a better-defined front-to-back ratio. This improvement can be attributed to the suppression of surface waves excited in the dielectric substrate, which in turn leads to better sidelobe levels. This effect is consistently observed across the whole operating frequency range from 26 GHz to 34 GHz. In particular, when comparing the performance of Port 1 and Port 2, both with and without the AMC surface, the improvement in the antenna performance with the AMC is noticeable. The presence of the AMC layer effectively reduces backward radiation (improving front-to-back ratio) and increases the strength of the signal in the desired direction (gain), which is critical for the antenna efficiency and directivity.

## 4. Measurement Results and Discussion

### 4.1. Fabrication and Measurement

**Figure 10:**
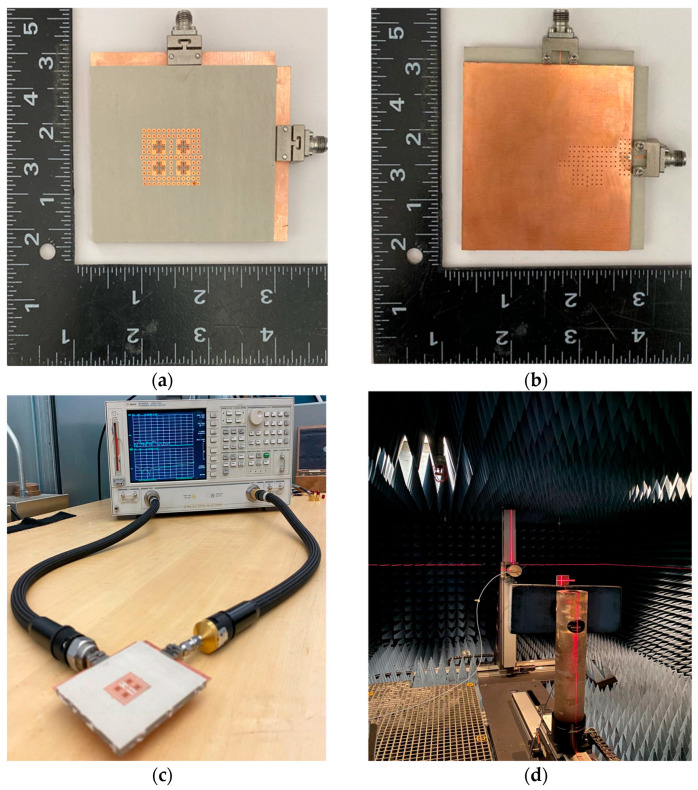
Fabricated prototype of the dual-polarized antenna array. (**a**) Top view, (**b**) bottom view, (**c**) the measurement setup for reflection coefficient characteristics, and (**d**) the measurement setup for the radiation pattern.

The proposed dual-polarized antenna array, optimized for IoT applications, was fabricated using printed circuit board (PCB) technology to validate its performance. The top and bottom views of the fabricated antenna array are shown in Figure 10a and Figure 10b, respectively. The layers of the antenna were bonded using epoxy under high temperature and pressure to ensure precise alignment and robust assembly.

**Figure 11:**
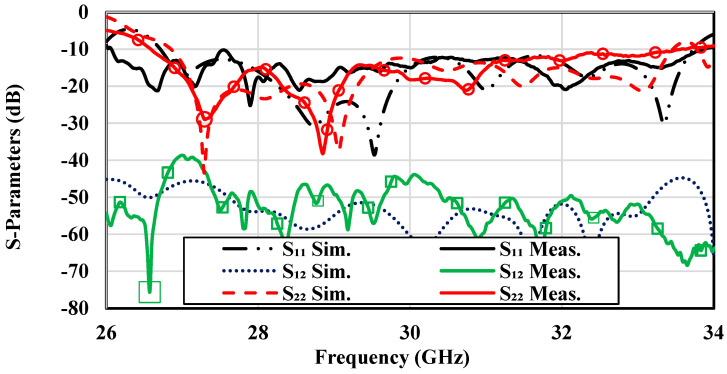
Simulated and measured S-parameters of the dual-polarized antenna array prototype.

To support testing, a microstrip line to PRGW transition was designed, achieving a matching level of −20 dB. The 50-Ω microstrip lines on the second and third layers were extended to connect to 2.92 mm (K) connectors, facilitating reliable signal transmission and measurements critical for IoT performance evaluations. The reflection coefficient characteristics were measured using an Agilent 8722ES Vector Network Analyzer (Agilent Technologies, Santa Clara, CA, USA), with the measurement setup illustrated in Figure 10c. Figure 11 compares the simulated and measured S-parameters, revealing an overlapped relative bandwidth of 24% within the frequency range of 26.7–33.7 GHz. The antenna achieved matching levels better than −10 dB and high isolation above 40 dB between dual polarizations across the operational band, ensuring reliable communication for IoT devices.

**Figure 12:**
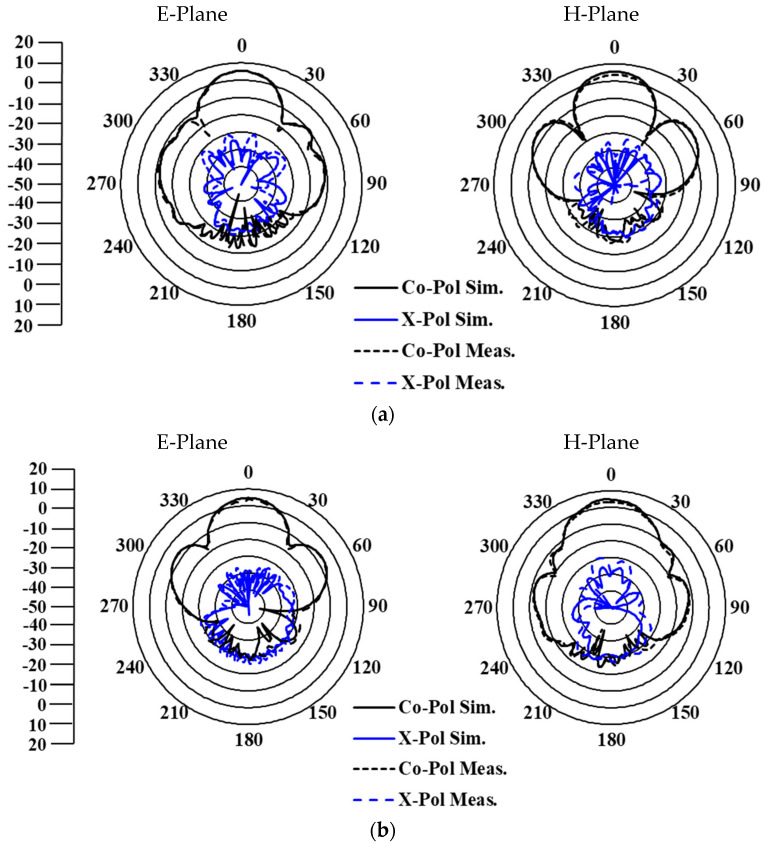
Simulated and measured radiation patterns of the dual-polarized antenna array at 30 GHz for (**a**) Port 1 and (**b**) Port 2.

Radiation pattern and gain measurements were conducted in an anechoic chamber, as shown in Figure 10c. The measured and simulated results for co-polarization (CO-Pol.) and cross-polarization (X-Pol.) in the E- and H-planes at 30 GHz are shown in Figure 12. Both sets of results exhibit stable, symmetric radiation patterns with low cross-polarization levels (−20 dB) in the boresight direction, essential for minimizing interference in IoT networks.

Minor discrepancies between the measured and simulated results can be attributed to several practical factors inherent in the fabrication and measurement processes. These include typical fabrication tolerances such as small deviations in substrate thickness, slight misalignment or positioning errors during layer assembly, and variations in via placement. Additionally, material inconsistencies, such as minor differences in dielectric constant or loss tangent from the manufacturer’s nominal values, may also contribute to the observed differences. Despite these factors, the close agreement between simulation and measurement demonstrates the robustness of the proposed antenna design and validates its practical applicability.

**Figure 13:**
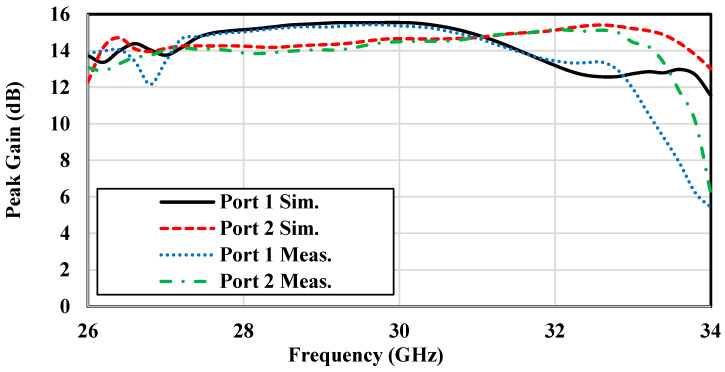
Simulated and measured peak gain of the dual-polarized antenna array prototype.

**Figure 14:**
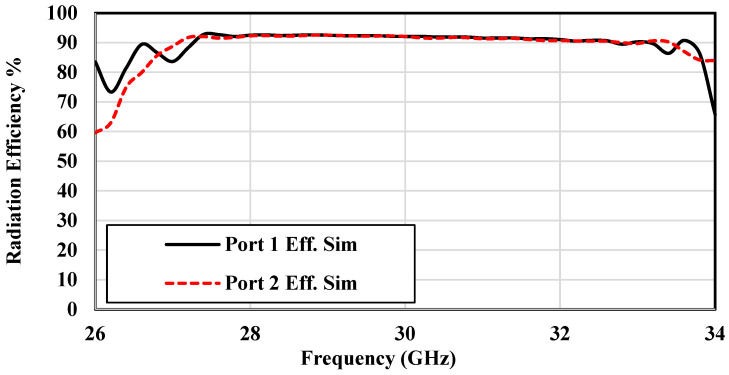
Simulated radiation efficiency of the dual-polarized antenna array prototype.

Figure 13 and Figure 14 present the gain and efficiency performance of the antenna across the operational frequency range. Both measured and simulated results show consistent gain values of approximately 13 dBi ± 0.5 dBi, with Port 1 reaching a peak gain of 13.64 dB and Port 2 achieving 13.9 dB. Figure 14 highlights simulated radiation efficiency exceeding 90% across the band, indicating exceptional performance suitable for high-efficiency IoT applications. These results confirm the antenna capability to support the reliable and efficient data transmission demands of next-generation IoT systems.

### 4.2. Comparison and Discussion

This section compares the performance of the proposed dual-polarized antenna array with other designs that utilize various guiding structures, as summarized in Table 2. The comparison systematically analyzes attributes such as gain, bandwidth, isolation, and fabrication techniques to underline the advantages of the proposed design for Internet of Things (IoT) applications. The proposed design, featuring a 2 × 2 ME-dipole antenna array with PRGW feeding and PCB fabrication technology, operates at 30 GHz and achieves an impedance bandwidth of 24%, isolation above 40 dB, and a gain of 13.88 dBi. This setup not only provides a competitive edge in cost-effectiveness and manufacturability but also addresses the higher losses typically associated with other technologies like SIW at millimeter-wave frequencies, as used in the designs of references [46,57]. While the ME-dipole arrays in these references show promising results, the proposed design achieves better bandwidth and lower losses.

Particularly, both the ME-dipole 1 × 8 array in reference [44] and the PRGW-fed 1 × 4 ME-dipole antenna array in [53] demonstrate significant gains of 16.1 dBi and 26.5, respectively. However, these gains come at the expense of a larger antenna structure in [44] and through a four-layer 3 × 3 SRR lens in [53], which may not be practical for all IoT applications where space and profile are at a premium. This may sacrifice the low-profile advantage necessary for practical deployment. 

In contrast, the hollow waveguide-fed antenna in reference [58] achieves a very higher gain of 26.5 dBi due to its efficient waveguiding and reduced radiation loss. However, its reliance on CNC fabrication introduces significant cost and complexity. Similarly, the LTCC-based design in [42], while effective, involves high fabrication costs and complexity. In contrast, the PCB fabrication method employed in this work balances high performance with affordability and ease of production, making it a practical choice for IoT applications.

Compared to the single dual-polarized ME-dipole antenna presented in [54], the proposed antenna array offers superior gain and isolation. Furthermore, many of the antennas listed in Table 2 suffer from low isolation, emphasizing the effectiveness of the proposed design in achieving a harmonious balance between performance, manufacturability, and cost. This positions the antenna as a robust solution for the high-efficiency demands of next-generation IoT systems.

## 5. Conclusions

The dual-polarized antenna array has been developed in this study, marking a significant advancement in designing high-frequency antennas for IoT applications. Optimized for mm-wave frequencies, the proposed antenna has been specifically tailored for the demands of the 5G spectrum, where dual-polarization is critical in enhancing channel capacity and reliability. Through PRGW technology, the antenna effectively minimizes parasitic losses and maximizes efficiency, offering substantial gains and superior isolation compared to traditional designs. The aforementioned features of the proposed antenna array, including the significant gain and bandwidth coverage required by the IoT network, ensure efficient data transmission in a wide variety of IoT systems. Since IoT networks are usually the communication platform for extremely complex networks with a large number of devices, such as in smart cities, or sometimes sensitive smart applications such as healthcare monitors, they require an antenna technology that can handle this high density of devices and high sensitivity without any signal interference. The proposed antenna ensures clear and distinct communication channels by providing high isolation levels of over 40 dB between ports and different polarizations, effectively mitigating cross-talk between transmitted and received signals and minimizing signal interference. The high performance of the antenna array in gain, bandwidth, and isolation makes it a good candidate for future IoT applications, which require robust and efficient communication links to support the high throughput and low latency demands of modern systems. Addressing challenges related to future IoT applications through 5G connectivity, the dual-polarized antenna array not only satisfies but far exceeds the requirements of current wireless networks, promising enhanced communications in upcoming 5G-enabled networks.

## Figures and Tables

**Table 1:** Optimized dimensions of the proposed CP ME-dipole antenna (units: mm).

Parameters	*e*_1_	*d*_1_	*w_a_*_1_	*w_c_*_1_	*w_d_*_1_	*w_t_*_1_ = *w_t_*_2_	*s*
Values (mm)	0.34	1.68	0.72	4.97	0.81	1.37	0.89
Parameters	*k_c_*_2_	*k_d_*_2_	*s_b_*_2_	*l_c_*_2_	*w_s_*_2_	*w_r_*_2_	*w_d_*_2_
Values (mm)	2.65	3.46	0.8	4.66	1.51	1.99	1.57
Parameters	*s_a_*_3_	*l_c_*_3_	*l_b_*_3_	*l_a_*_3_	*d_a_*_4_	*l_a_*_4_	*w_a_*_4_
Values (mm)	1.38	6.01	2.89	6.5	3.97	2.45	0.54

**Table 2:** Comparison of the proposed dual-polarized antenna array with other reported mm-wave dual-polarized designs in the literature.

Ref.	Radiating Element	Array Scale	Feeding Technology	Fabrication Technology	*F*_0_ (GHz)	Imp. Bandwidth (−10 dB)	Isolation Level (dB)	Gain (dBi)
[46]	ME-dipole	2 × 2	SIW	PCB	60	22%	>15	12.5
[57]	ME-dipole	1 × 8	SIW	PCB	60	21%	>45	16.1
[53]	ME-dipole + Lens	1 × 4	PRGW	PCB	31.5	22%	N. A	16
[58]	Waveguide	8 × 8	HW	PCB + CNC	19.7	17.4%	>14	26.5
[42]	Patch	4 × 4	L-Probe	LTCC	26.8	19.5%	>22	12.7
[54]	ME-dipole	1 × 1	PRGW	PCB	30	23.4%	>20	10.5
This work	ME-dipole	2 × 2	PRGW	PCB	30	24%	>40	13.88

## Data Availability

The data presented in this study are openly available.
